# Research and Verification of a Novel Interferometry Method by Joint Processing of Downlink Pseudo-Noise Ranging and DOR Signals for Deep Space Exploration

**DOI:** 10.3390/s24030822

**Published:** 2024-01-26

**Authors:** Weitao Lu, Min Fan, Lue Chen, Dezhen Xu, Yujia Zhang, Tianpeng Ren

**Affiliations:** 1 Beijing Aerospace Control Center, Beijing 100094, China; chenlue@xao.ac.cn (L.C.); zhangyujia17@mails.ucas.ac.cn (Y.Z.); shemmer@126.com (T.R.); 2 Beijing Institute of Tracking and Telecommunications Technology, Beijing 100094, China; xudezhen@bittt.cn

**Keywords:** deep space exploration, power of signals, regenerative pseudo-noise ranging, interferometry measurement, integer ambiguity resolution, QueQiao-1 relay satellite of lunar

## Abstract

The remarkably long distances covered by deep space probes result in extremely weak downlink signals, which poses great challenges for ground measurement systems. In the current climate, improving the comprehensive utilization of downlink signal power to increase the detection distance or enhance the measurement accuracy is of great significance in deep space exploration. Facing this problem, we analyze the delta Differential One-way Range (ΔDOR) error budget of the X-band of the China Deep Space Network (CDSN). Then, we propose a novel interferometry method that detunes one group of DOR beacons and reuses the clock components of regenerative pseudo-code ranging signals for interferometry delay estimation. The primary advantage of this method is its ability to enhance the power utilization efficiency of downlink signals, thereby facilitating more efficient tracking and measurement without necessitating additional design requirements for deep space transponders. Finally, we analyze and verify the correctness and effectiveness of our proposed method using measured data from CDSN. Our results indicate that the proposed method can save approximately 13% of the downlink signal power and increase the detection distance by about 6.25% using typical modulation parameters. Furthermore, if the relative power of other signal components remains unchanged, the power of the DOR tone can be directly increased by more than 100%, improving the deep space exploration ability more significantly.

## 1. Introduction

At present, the primary method employed by major space agencies globally for tracking probes in lunar and deep spaces is ground-based radio measurement technology, which is particularly critical for key tracking arcs such as transfer orbit correction, capture, and the landing of the target celestial body. The ground-based radio tracking system for deep space typically provides measurements of ranging, range-rate, and interferometry delay. According to the Consultative Committee for Space Data Systems (CCSDS) specification, a Tracking Telemetry and Command (TT&C) transponder with unified carrier modulation is usually configured on the probes, transmitting the uplink signal back to the ground station. During this process, the TT&C transponder generates and modulates the ranging signals and Differential One-way Ranging (DOR) beacon to the downlink carrier [1]. The ground station demodulates the received downlink signals to achieve ranging and interferometry delay measurements, which can be used for orbit determination and the navigation of the lunar and deep space probes.

Side-tone ranging, tone-code hybrid ranging, and regenerative pseudo-noise (PN) code ranging systems are widely used in the realm of deep space exploration. The Chang ‘E series lunar exploration missions conducted by China, as well as TianWen-1, the first Mars exploration mission, both employ the side-tone ranging system, achieving an accuracy of better than 1 m at the X-band [2]. The main ranging system for deep space probes of the National Aeronautics and Space Administration (NASA) and European Space Agency (ESA) is a tone-code hybrid ranging system with an accuracy of 1 m. However, it is noteworthy that both the side-tone and tone-code hybrid ranging systems are operated in the turn-around mode. This means that while the ranging signal is transmitted, the received noise and residual command signals are also turned around. This is not suitable for deep space exploration with longer distances and weaker downlink signals [3]. The downlink signal of the regenerative PN code ranging system is more suitable for deep space exploration contexts due to its absence of forwarding noise, coupled with a substantial unambiguous range and heightened measurement accuracy [4,5]. The Chinese QueQiao-1 lunar relay satellite has carried out a regenerative pseudo-noise ranging experiment in orbit, improving the ranging accuracy by an order of magnitude compared with side-tone ranging [6].

Very long baseline interferometry (VLBI) is one of the main techniques for tracking and navigating lunar and deep space probes. In the 1970s, the NASA Deep Space Network (DSN) verified the navigation and orbit determination of the Voyager 1 and Voyager 2 spacecraft using VLBI. From the 1970s to the 1980s, the VLBI measurement accuracy for the interplanetary missions of NASA reached an impressive 30 nrad. In 2001, the accuracy of VLBI reached about 5 nrad in Mars Odyssey and further increased to 2 nrad in the Mars Reconnaissance Orbiter (MRO) exploration [7]. The Deep Space Network of the former Soviet Union consisted of three deep space stations with a 64 m antenna in Bear Lake near Moscow, a 70 m antenna in Ivpatoria in Crimea, Ukraine, and a 70 m antenna in Ussurisk. At present, the tracking and measurement of Russian deep space probes are mainly carried out by the Bear Lake and Ussurisk deep space stations. In the 1980s and 1990s, the Russian Deep Space Network conducted surveys of probes such as Venus-15 and Vega-1 [8]. ΔVLBI measurement experiments were also conducted on celestial bodies in the solar system, such as Venus and Mars. The ΔVLBI measurements were derived from the interference fringes of the radar echo [9]. However, in recent years, there has been no successful implementation of lunar and deep space exploration in Russia. Despite this, it is anticipated that they will continue their lunar exploration activities in the future. In China, VLBI has been extensively applied to the Chang’E series lunar explorations and its first Mars exploration. VLBI technology was first verified using the TT&C signal of the Chang’E-1 spacecraft with a bandwidth of about 1 MHz [10]. The Differential One-way Range (DOR) signals were set up according to the CCSDS specification in the Chang’E-2 mission [11]. The X-band ΔDOR on-orbit experiment was conducted, achieving an interferometry delay accuracy of about 0.5 ns [12]. The Same-Beam Interferometry (SBI) experiment was conducted during the Chang’E-3 mission, yielding a post-processing differential phase delay accuracy of approximately 1 pico-second. On the basis of these measurement data, the relative positioning accuracy of the Chang’E-3 lunar probe reached up to roughly 1 m [13,14,15]. Subsequent experiments involving multi-probe and multi-signal interferometry technology were executed in Chang’E-4 and Chang’E-5. The accuracy of orbit determination was increased to the order of hundreds of meters by the comprehensive utilization of ranging, range-rate, and VLBI interferometry delay measurement for trans-earth or trans-lunar trajectory [16,17,18,19,20,21]. In the Tianwen-1 exploration, the average error of the ΔDOR time-delay was about 0.11 ns [22], and the positioning accuracy of the rover was up to 100 m [23].

However, with the ongoing advancement of deep space exploration, the distance between explorers and ground stations is progressively increasing, which leads to significantly weak downlink signals, thereby limiting the measuring performance of ground-based systems. The primary challenges in deep space exploration include enhancing the comprehensive utilization of downlink signal power to extend the exploration distance and improve the measurement accuracy. In the realm of deep space radio measurement, the predominant technical methods for improving weak signal processing capabilities encompass regenerative pseudo-code technology and antenna array technology. Additionally, it is useful to increase antenna aperture, transmission power on the spacecraft, and integration time. At present, the maximum aperture of the commonly used antenna in deep space exploration is 70 m. Any further enlargement of the aperture would substantially escalate the system construction costs. The increase in transmission power on the spacecraft is limited by current manufacturing levels. While the increase in integration time can improve the influence of thermal noise, it is constrained by the accuracy of the probe’s predicted ephemeris. Antenna array technology is generally used to improve the performance of data transmission in the communication field. Due to the requirement of radio measurement for the system’s phase center, the combined signal of an antenna array is difficult to use directly for measurement. Regenerative pseudo-code technology was originally designed to improve the ranging accuracy in the field of deep space exploration. It simultaneously improves the measurement accuracy and resolves the ambiguity problem associated with ranging. In the current trend of signal multiplexing in deep space exploration, the regenerative pseudo-code technology can be combined with traditional radio measurement technology to enhance the weak signal processing capability of the system.

CCSDS specifications recommend that the frequency spacing between the two sets of DOR beacon signals and the carrier in the X-band downlink signal is approximately ±3.8 MHz (denoted as ±DOR_1_) and ±19.2 MHz (denoted as ±DOR_2_), respectively [24,25]. Typically, DOR_1_ signals are employed to resolve time delay ambiguity while DOR_2_ signals facilitate high-precision interferometric time delay acquisition. The clock tone frequency of the regenerative pseudo-noise is roughly ±0.5 MHz (1 Mchips/s) or ±1 MHz (2 Mchips/s) from the downlink carrier, resulting in an effective bandwidth of 1 MHz or 2 MHz, which closely matches that of the DOR_1_ beacon signal. Therefore, it becomes feasible to simultaneously achieve regenerative pseudo-noise ranging and ΔDOR [26]. To this end, a novel interferometry processing method that combines pseudo-noise ranging and DOR signals is presented. This approach employs the pseudo-noise ranging clock component to solve the interferometry delay ambiguity, subsequently deriving the final interferometry delay through the joint processing of the downlink carrier and DOR_2_ beacon signals. Notably, this innovative method repurposes the regenerated pseudo-code ranging signal, facilitating the integrated application of both ranging and DOR beacon signals, thereby enhancing the comprehensive utilization rate of the downlink signal power. In the case of the limited downlink signal power of spacecraft, the proposed method may improve the measurement accuracy or enlarge the detection distance, providing direct technical support for the efficient use of downlink signal power and for the design of a TT&C downlink signal structure in a weak-signal scenario such as interplanetary deep space exploration.

This paper is structured as follows. Section 2 delineates the fundamental principle of ΔDOR, a prevalent VLBI technology system, and provides an analysis of its error budget. Section 3 offers a comprehensive presentation of the downlink signal spectrum structure of spacecraft and the proposed method. And Section 4 analyses the performance of the proposed method, contrasting it with traditional methods for comparison. Subsequently, Section 5 further validates the proposed method on the basis of measured interferometry data from the China Deep Space Network (CDSN). Finally, the conclusions are presented in Section 6.

## 2. Problem Description

### 2.1. The Basic Principle of ΔDOR

**Figure 1:**
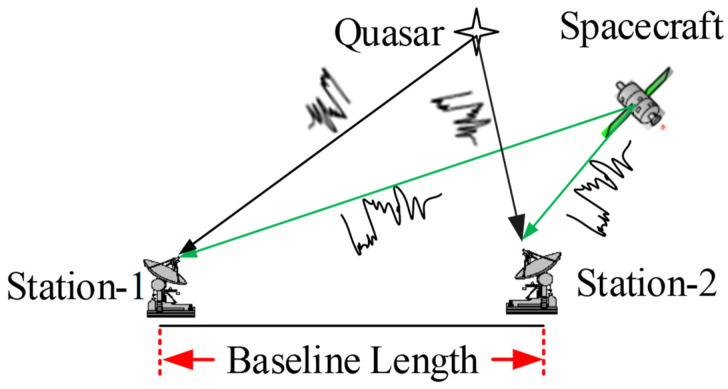
The basic principle of ΔDOR.

ΔDOR is one of the commonly used VLBI technology systems, the fundamental principle of which is illustrated in Figure 1. Two distant stations, denoted as Station-1 and Station-2 in Figure 1, establish an interferometry baseline, with the distance between them referred to as the baseline length. The two stations simultaneously observe the spacecraft or the reference radio source alternately during a ∆DOR measurement. The radio source measurement serves to eliminate the system’s linear error, thereby facilitating the acquisition of precise interferometry time delay measurements for the spacecraft.

Usually, the quasar position is defined in the celestial reference system, while the station or baseline coordinates are established in the terrestrial reference system. Consequently, during data processing, it is necessary to transform these coordinates from the terrestrial to the celestial reference system, where the Earth Orientation Parameters (EOP) including polar motions, the difference between Universal Time-1 and Coordinated Universal Time (UT1-UTC), nutation, and precession will be used. It is evident that the precision of the EOP also has an effect on the accuracy of the ΔDOR measurements.

### 2.2. Error Budget Analysis

The potential sources of error in a ∆DOR measurement encompass the received tone power-to-noise ratio and the effective bandwidth of the DOR tones, both of which contribute to random errors. Other error sources include the precision of the quasar delay measurement, the quasar position accuracy, the clock stability error, the instrumental phase ripple, the uncertainties in the Earth Rotation Parameters and atmospheric media delay error, each of which can potentially induce systematic errors. References [27,28] have conducted an analysis on the typical influence of the above errors of Mars exploration in the Deep Space Network (DSN).

At present, the fields of radio measurement technology and geodetic measurement technology are experiencing rapid advancement. As orbit prediction becomes increasingly precise, the integration time for correlation processing can extend to a magnitude of up to 10 s in CDSN interferometry systems. The equipment used for data recording and acquisition has evolved, allowing for a higher interferometry data sampling frequency. The accuracy of station coordinates has been improved to the mm level, and the prediction accuracy of UT1-UTC has also been improved to reduce the influence of Earth orientation error. Quasars selected as ∆DOR reference sources possess positions that are recognized in the International Celestial Reference Frame (ICRF), where most catalog sources exhibit a positional accuracy better than 0.3 nrad [28]. For ∆DOR, it is beneficial to select compact sources with an accurate position. Research results have shown that the troposphere zenith delay measurement accuracy has been improved to about 5 mm [28]. Thus, an analysis of the ∆DOR measurement error effects in the CDSN system is given.

The distance from the spacecraft to the receiver is about 150 million km, and the radio frequency of the downlink signal is 8.4 GHz. The effective transmitted tone power along the line-of-sight to the receiver is 40 dBW, and the modulation loss is about 21 dB. The diameters of the two antennas on the baseline are 65 m and 35 m, and their efficiencies and system noise temperature are both 0.55 and 50 K [29]. According to the CCSDS standard, the total spanned bandwidth is set to 38.25 MHz. The integration time of spacecraft and quasar signal correlation processing is 30 s and 300 s, respectively. The system loss factor stands at 0.8, which can vary depending on the implementation. The number of time-multiplexed frequency channels is four. The quasar position uncertainty in ICRF3 is about 0.2 nrad, and the number of quasar data samples per second is 8 × 10^6^ (corresponding to 8 MHz sample frequency). The projected baseline length is about 4000 km. The uncertainties in the baseline coordinates and orientation are 1 cm and 3 cm, respectively. The clock instability is 10^−14^ and the instrumental phase ripple is 0.2 degrees. The elevations of the spacecraft and quasar are 20 degrees and 25 degrees, respectively, and their angular separation is 10 degrees. The zenith troposphere delay uncertainty is 0.5 cm. The sun–earth–source angle is 50 degrees, and the solar wind velocity is 400 km/s.

**Figure 2:**
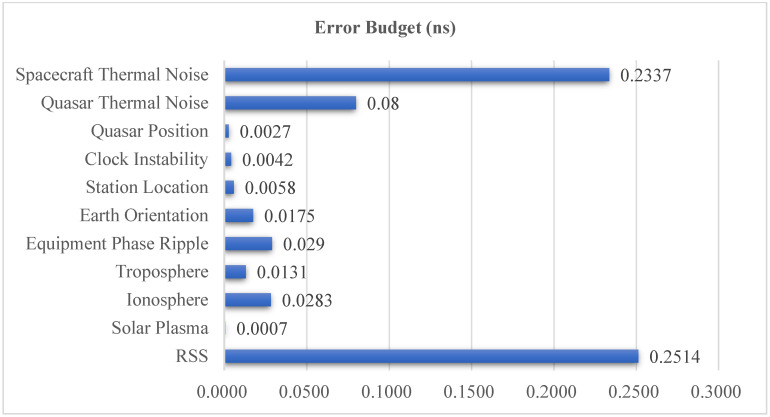
ΔDOR error budget for X-band in CDSN interferometry system.

The error budget for the X-band ΔDOR in the CDSN interferometry system with the above conditions is shown in Figure 2, where RSS stands for the Root Sum Square of all the above ten errors and is always used as a system’s total error criterion. As is evident from Figure 2, spacecraft thermal noise emerges as the predominant error source, accounting for approximately 93% of the overall error effect. The thermal noise error, which is believed to be a random error source, can be calculated using Formula (1) [27,28], where Δ*f* represents the effective bandwidth, and hardly changes under the CCSDS specification, and *T* is the integration time, *P*/*N*_0_ being the carrier-to-noise power density ratio, which is related to the downlink signal power. Consequently, primary strategies to eliminate thermal noise encompass enhancing the effective bandwidth, increasing the integration time, and optimizing the carrier-to-noise ratio. Notably, the effective bandwidth is limited by the CCSDS standard; specifically, the S-band and X-band effective bandwidths for VLBI are approximately 7.5 MHz and 40 MHz, respectively. The carrier-to-noise ratio is related to the downlink signal power and the receiving performance of the ground station. Since the receiving performance of the ground station is usually stable and has little room to improve, the carrier-to-noise ratio is directly affected by the downlink signal power.

(1) $$\sigma_{\tau }=\frac{\sqrt{2}}{2\pi \Delta f\sqrt{T(P/N_{0})}}$$

The integration time is directly limited by the accuracy of the spacecraft’s predicted ephemeris. The data processing of interferometry is essentially the correlation of two station data which have been compensated for by using a prior time delay model and offers the arrival time difference of the same wavefront. The prior time delay model is calculated by using the spacecraft’s predicted ephemeris and the station’s coordinates. Inaccurately predicted ephemeris can lead to an imprecise prior delay model, resulting in a significant residual delay rate during correlation processing. Suppose the residual delay rate is *f*_r_ and the integration time is *T*, only when *f*_r_ × *T* is far less than 1, can the correlation peak of the two station data be close to the maximum value. However, if the predicted ephemeris is inaccurate, leading to a large *f*_r_, then an increase in the integration time results in a decrease in both the correlation peak and measurement accuracy. In other words, the increase in the integration time is constrained by the accuracy of the predicted ephemeris.

Therefore, while holding other parameters fixed, improving the utilization efficiency of the downlink signal power, which equivalently increases the downlink signal power-to-noise, will be beneficial to improve the measurement accuracy, especially in the far deep space explorations.

## 3. Materials and Methods

### 3.1. Downlink Signal Spectrum Structure

**Figure 3:**
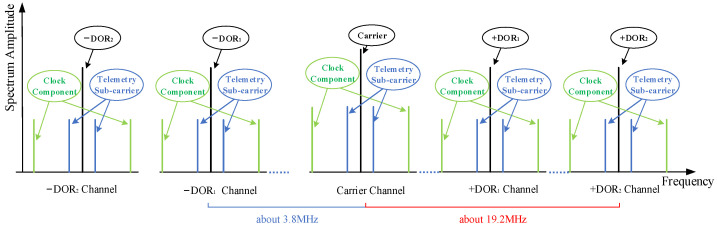
The X-band downlink signal spectrum construction of a typical deep spacecraft.

Currently, recommended as ranging pseudo-codes by the CCSDS [30], T4B and T2B are both generated by combining six identical codes with different logistics. The primary distinction between these two lies in that the weight of the clock component of the T4B code is double that of the T2B code. The frequency of the PN code ranging clock tone component is about 0.5 MHz or about 1 MHz when the PN code rate is 1 Mchips/s or 2 Mchips/s. In addition, according to the CCSDS standard, only one group of DOR beacon signals is set in the S-band, and the frequency spacing between the DOR signal and the carrier is 1/600 of RF frequency, which is about 3.7 MHz when the RF frequency is 2.25 GHz. Two sets of DOR beacon signals are allocated in the X-band, and the frequency spacings are 1/2200 (corresponding to DOR_1_) and 1/440 (corresponding to DOR_2_) of RF frequency, respectively, which are about 3.8 MHz and 19.2 MHz when the radio frequency is 8.45 GHz. The X-band downlink signal spectrum construction of a typical deep spacecraft is shown in Figure 3.

### 3.2. Proposed Method

The processing of very long baseline interferometry data typically involves three steps: initial delay model compensation, correlation, and frequency synchronization. First, the initial time delay model is calculated by using the target’s prior ephemeris and station coordinates, which is to compensate the raw data recorded at the stations. Then, the original data after compensation is correlated to obtain the cross-spectrum and interferometry fringes. Finally, frequency synchronization is performed to estimate the high-precision interferometry time delay. The initial cross-phases of the DOR beacons are typically located in the range of [−π, π). Since the time delay model is usually not accurate enough, there is integer ambiguity at the cross-spectrum phases of ±DOR_2_, and even of ±DOR_1_, which implies that the initial cross-phases of ±DOR_2_ and ±DOR_1_ should be corrected by integer times of 2π. As a result, the ambiguity should be resolved by using coarse time delay estimation that is unambiguous before the final delay estimation.

**Figure 4:**
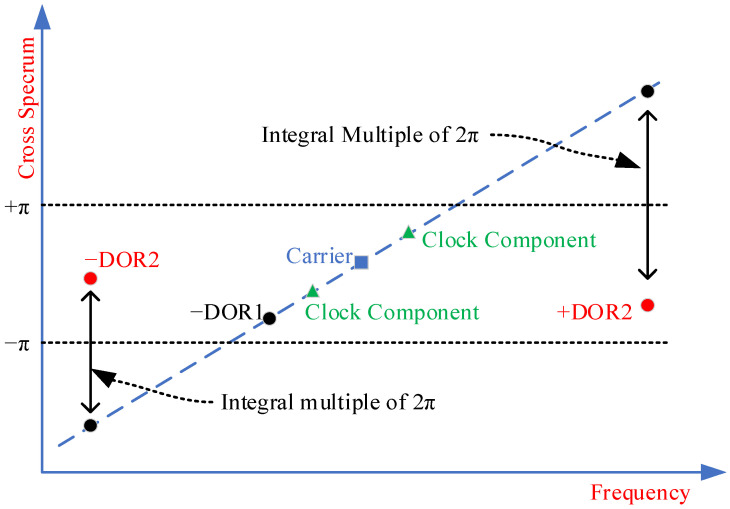
The cross-phase ambiguity resolving of X-band downlink signal in PN code ranging system.

Taking the X-band as an example, the four beacons including the downlink carrier, ±DOR2, −DOR_1,_ or +DOR_1_ are usually sampled and recorded for VLBI data processing. First, the time delay is coarsely estimated by using the downlink carrier and one of the ±DOR_1_ beacons to resolve the integer ambiguity of the cross-phase of the ±DOR_2_ signal. Second, the cross-phase of the ±DOR_2_ signal undergoes integer times of 2π correction. Finally, the frequency synchronization is conducted by jointly using the four beacons to obtain the high-accuracy time delay estimation. As shown, the characteristics of the pseudo-code clock component signal are quite consistent with that of the DOR beacons. Its frequency space away from the carrier is close to the S-band DOR beacon or the X-band DOR_1_ beacon. Based on these considerations, we propose employing the clock component of the pseudo-code signal and carrier to roughly estimate the time delay in order to resolve potential integer ambiguity in the cross-phase of the ±DOR2 beacons, thereby achieving precise time delay estimation. The ambiguity resolving process of the X-band is shown in Figure 4, and the detailed process can be found in references [7,31].

Suppose that *f*_1_, *f*_2_ correspond to the two frequencies of the signals in Figure 3, for instance, the carrier and −DOR_1_ signal, and *φ*_1_, *φ*_2_ are the corresponding cross-spectrum phases, then, the time delay can be estimated by Formula (2):

(2) $$\hat{\tau }=\frac{\varphi_{2}-\varphi_{1}}{2\pi \left(f_{2}-f_{1}\right)}$$

During the data processing, the cross-spectrum phases are normalized to the main value zone, [−π, π). If there is ambiguity in the cross-spectrum phase, the estimated time delay will be biased. Therefore, one of the preconditions before frequency synchronization is that the cross-spectrum phases of the ±DOR1 signal have no ambiguity. The process of resolving the time delay ambiguity in VLBI imposes stringent requirements on the accuracy of the prior time delay model [21], which almost equals half of the reciprocal of the ambiguity resolving signal bandwidth. For the X-band, the ambiguity resolving signal is usually the DOR_1_ beacon, and the corresponding signal bandwidth is about 3.8 MHz. Accordingly, the prior time delay model should be more accurate than about 130 ns. Suppose the chip rate of the regenerated pseudo-code ranging signal is 2 Mchips/s, the frequency interval between the clock component of the pseudo-code ranging signal and the carrier will be about ±1 MHz, and consequently, the prior time delay model should be more accurate than about 500 ns. In other words, the accuracy requirement of the delay model for integer ambiguity resolution using the clock component of the pseudo-code ranging signal can be relatively reduced.

Based on the partial derivation of Formula (2), the relationship between the accuracy of the time-delay estimation and the performance of the cross-phase estimation can be obtained, expressed as Formula (3):

(3) $$\sigma_{\hat{\tau }}=\frac{\sqrt{2}}{2\pi \left(f_{2}-f_{1}\right)}\sigma_{\varphi }=\frac{\sqrt{2}}{2\pi \Delta f}\sigma_{\varphi }$$

where Δ*f* means the effective bandwidth, and $\sigma_{\varphi }$ and $\sigma_{\hat{\tau }}$ are the accuracies of the cross-phase and interferometry delay, respectively. Formula (3) indicates that the wider the effective bandwidth is, and the more accurately the cross-phase is estimated, the more accurate the time delay estimation will be. According to the power allocation of the downlink signal, the power of the pseudo-code clock component is slightly stronger than that of the DOR_1_ signal, but with a narrower bandwidth. Therefore, the precision of the estimated time delay by using the clock component of the pseudo-code ranging signal may be worse than that of using the −DOR_1_ or +DOR_1_ signal.

On the basis of Formula (2), the integer ambiguity of the cross-phase of the ±DOR_2_ signal can be obtained.

(4) $$N_{\mathrm{ambi}}=\left[f_{\mathrm{DOR}2}\hat{\tau }\right]$$

where [*x*] denotes the integer value closest to *x*. The coarsely estimated time delay can be expressed as Formula (5), where τ denotes the real time delay and Δτ is the estimation bias. Usually, Δτ can be modeled as a stochastic variety, the mean square error of which represents the time delay accuracy in Formula (3).

(5) $$\hat{\tau }=\tau +\Delta \tau$$

According to the 3σ rule, the delay estimation bias, Δτ, will fall into the value zone of $\left[\tau -3\sigma_{\hat{\tau }},\tau +3\sigma_{\hat{\tau }}\right]$ with a probability of 99.7%, which can be described using Formula (6):

(6) $$P\left(\left|\Delta \tau \right|\leq 3\sigma_{\hat{\tau }}\right)=99.7\%$$

Therefore, in order to ensure the correctness of the integer ambiguity resolution, the accuracy requirement of the time delay estimation can be obtained from Formulas (4)–(6):

(7) $$\sigma_{\hat{\tau }}<\frac{1}{6f_{\mathrm{DOR}2}}$$

The accuracy requirement of the cross-phase estimation can be obtained by combining Formulas (3) and (7):

(8) $$\sigma_{\varphi }<\frac{\pi \Delta f}{3\sqrt{2}f_{\mathrm{DOR}2}}$$

It can be seen that the wider the effective bandwidth, the lower the requirement of the phase estimation accuracy. Therefore, the cross-phase estimation accuracy of the clock component should be higher than that of the −DOR_1_ or +DOR_1_ signal when the integer ambiguity is resolved. For the two groups of the X-band DOR beacon signals, Δ*f* = 3.8 MHz, *f*_DOR2_ = 19.2 MHz, the phase estimation accuracy is not less than 0.1466 rad. Suppose the pseudo-code rate is 2 Mchips/s, its clock component is 1 MHz away from the carrier, that is, Δ*f* = 1 MHz, and *f*_DOR2_ remains unchanged, the corresponding phase estimation accuracy is no worse than 0.0386 rad.

In summary, using the X-band data processing as a representative example, when the pseudo-code ranging signal (±PNc) and DOR beacon signal are processed jointly, the flow of the proposed interferometry method is as follows:

Step 1: sample and record the four signals including −DOR_2_, −PNc or +PNc, carrier, and +DOR_2_.
Step 2: compensate the raw data recorded on the stations by using the initial delay model, and then conduct correlation to obtain the cross-phases of the four signals in Step 1.
Step 3: obtain the coarse time delay estimation by jointly processing the −PNc or +PNc signal and the carrier signal.
Step 4: calculate the ambiguity of the cross-phase of the ±DOR_2_ signal by using the coarse time delay estimation from Step 3.
Step 5: modify the cross-phase of the ±DOR_2_ signal by integer multiples of 2π.
Step 6: synchronize the modified ±DOR_2_ signal, −PNc or +PNc, and carrier to estimate the high-precision interferometry delay.

The primary advantage of the proposed method is that when there is a pseudo-code ranging clock component, the DOR beacon used for ambiguity resolution in the detector downlink signal can be detuned, and furthermore, without any additional changes to deep space transponders, the downlink signal power configuration is adjusted to enhance the measurement signal power and to improve the measurement accuracy or increase the detection distance. The accuracy of interferometry delay measurement is proportional to the effective bandwidth. Under the same conditions, the wider the effective bandwidth, the higher the accuracy of the time delay measurement, yet the problem is that the ambiguity of the time delay becomes smaller. According to the CCSDS standard, two groups of DOR beacons are set for the X-band, DOR_1,_ and DOR_2_. DOR_1_ primarily resolves the time delay ambiguity, while ±DOR_2_ ensures measurement accuracy. For the method proposed in this paper, when the pseudo-code clock component is used to replace the DOR_1_ signal, the effective bandwidth of the coarse time delay estimation becomes narrower. If the same time delay measurement accuracy is achieved, the cross-spectrum phase measurement accuracy must be improved, otherwise, the time delay measurement accuracy will deteriorate. However, as long as this does not result in integer ambiguity resolution errors, the final delay measurement accuracy aligns with traditional methods, because the ±DOR_2_ signal beacons remain unchanged. There is only one set of DOR beacons for the S-band, and it can be directly equivalent to the coarse delay estimation process in the X-band. When the DOR signal is used, the effective bandwidth is wider, and the measurement accuracy is higher than that of the pseudo-code clock component. Therefore, the method proposed in this paper is more suitable with a weak downlink signal power in the X-band.

## 4. Improvement Analysis of Downlink Signal Power Utilization Efficiency

Taking the X-band for example, the spacecraft downlink signal can be expressed as Formula (9) [27,32]. For the S-band signal, there is only one group of DOR beacons, and the DOR_2_ items should be removed from the following formula.

(9) $$s(t)=\sqrt{2P_{T}}\cos\left(\begin{array}{l} 2\pi f_{c}t+m_{\mathrm{TM}}x_{\mathrm{TM}}(t)+m_{\mathrm{RG}}x_{\mathrm{RG}}(t)+\\ m_{\mathrm{DOR}1}\sin(2\pi f_{\mathrm{DOR}1}t)+m_{\mathrm{DOR}2}\sin(2\pi f_{\mathrm{DOR}2}t) \end{array}\right)$$

As shown in Formula (9), $P_{T}$ is the total power of the downlink signal, $f_{c}$, $f_{\mathrm{DOR}1}$ and $f_{\mathrm{DOR}2}$ are the frequencies of the carrier and two groups of DOR beacon signals, respectively. $m_{\mathrm{TM}}$, $m_{\mathrm{RG}}$, $m_{\mathrm{DOR}1}$, and $m_{\mathrm{DOR}2}$ are the modulation degrees of the telemetry signal, ranging signal, and two sets of DOR beacon signals, respectively. Notably, a higher modulation degree corresponds to an increased downlink power. $x_{\mathrm{TM}}(t)$ and $x_{\mathrm{RG}}(t)$ denote the telemetry signal and ranging signal, the detailed information of which can be referred to in reference [30]. For the pseudo-code ranging system, the sinusoidal signal is usually one of the subcarrier types, and then the downlink power of the pseudo-code ranging signal and the DOR beacon signal can be expressed as follows:

(10) $$\left\{ \begin{array}{l} P_{\mathrm{RG}}=P_{T}\cos^{2}(m_{\mathrm{TM}})J_{1}^{2}\left(m_{\mathrm{RG}}\right)J_{0}^{2}\left(m_{\mathrm{DOR}1}\right)J_{0}^{2}\left(m_{\mathrm{DOR}2}\right)\\ P_{\mathrm{DOR}1}=P_{T}\cos^{2}(m_{\mathrm{TM}})J_{0}^{2}\left(m_{\mathrm{RG}}\right)J_{1}^{2}\left(m_{\mathrm{DOR}1}\right)J_{0}^{2}\left(m_{\mathrm{DOR}2}\right)\\ P_{\mathrm{DOR}2}=P_{T}\cos^{2}(m_{\mathrm{TM}})J_{0}^{2}\left(m_{\mathrm{RG}}\right)J_{0}^{2}\left(m_{\mathrm{DOR}1}\right)J_{1}^{2}\left(m_{\mathrm{DOR}2}\right) \end{array}\right.$$

Suppose the modulation degrees of the telemetry signal, ranging signal, and the two sets of DOR beacon signals are 0.8 rad, 0.6 rad, and 0.5 rad (for the two sets of DOR beacon signals, the modulation degrees are usually same), the corresponding ratio of the power of the pseudo-code ranging signal and the two sets of the DOR beacon signals to the total downlink power is about −15.09 dB and −16.80 dB, respectively.

The modulation of a downlink carrier signal by an increased number of signal types results in a more dispersed downlink signal power. If one of the modulated signals is detuned, the power of the remaining signals will increase even if the modulation degrees remain unchanged. Therefore, if the ±DOR_1_ beacon signal is not modulated to the downlink signal, the S/X band downlink signal can be expressed by Formula (11):

(11) $$\left\{ \begin{array}{l} s_{S}(t)=\sqrt{2P_{T}}\cos\left(2\pi f_{c}t+m_{\mathrm{TM}}x_{\mathrm{TM}}(t)+m_{\mathrm{RG}}x_{\mathrm{RG}}(t)\right)\\ s_{X}(t)=\sqrt{2P_{T}}\cos\left(\begin{array}{l} 2\pi f_{c}t+m_{\mathrm{TM}}x_{\mathrm{TM}}(t)+m_{\mathrm{RG}}x_{\mathrm{RG}}(t)+\\ m_{\mathrm{DOR}2}\sin(2\pi f_{\mathrm{DOR}2}t) \end{array}\right) \end{array}\right.$$

The power of the pseudo-code ranging signal and the DOR beacon signal in the X-band downlink signal can be expressed as Formula (12) when the ±DOR_1_ beacon signal is not modulated:

(12) $$\left\{ \begin{array}{l} P_{X,\mathrm{RG}}=P_{T}\cos^{2}(m_{\mathrm{TM}})J_{1}^{2}\left(m_{\mathrm{RG}}\right)J_{0}^{2}\left(m_{\mathrm{DOR}2}\right)\\ P_{X,\mathrm{DOR}2}=P_{T}\cos^{2}(m_{\mathrm{TM}})J_{0}^{2}\left(m_{\mathrm{RG}}\right)J_{1}^{2}\left(m_{\mathrm{DOR}2}\right) \end{array}\right.$$

Under the same condition of modulation as Equation (9), the ratio of the power of the pseudo-code ranging signal and the DOR_2_ beacon signal to the total power of the downlink signal are −14.54 dB and −16.25 dB, which are about 13% higher than that of the DOR_1_ beacon signal being modulated to the downlink carrier. If the power of the downlink ranging signal is unchanged and the power saved by detuning the DOR_1_ beacon signal is allocated to the DOR_2_ beacon signal, the power of the DOR_2_ beacon signal will increase by more than 100% (one times better) as illustrated in Table 1. 

Table 1 provides a detailed analysis of the scenarios. Scenario 1 stands for the traditional modulation scheme, while Scenario 2 and 3 exemplify the proposed method, using Scenario 1 as a reference. When the DOR_1_ beacon signal is not modulated in Scenario 2, that is, the modulation degree of the DOR_1_ beacon signal is set to 0 rad, the power of the DOR_2_ beacon signal is correspondingly larger by about 0.55 dB, which is about 13% (10^0.055^ ≈ 1.135) higher than that in Scenario 1. In Scenario 3, the DOR_1_ beacon signal is again not modulated and we enlarge the modulation degree of the DOR_2_ beacon signal until the power of the ranging signal equals that in Scenario 1, then, it can be seen that the power of the DOR_2_ beacon signal is correspondingly larger by about 3.2 dB, which is more than 100% higher compared with Scenario 1.

Similarly, the power of the pseudo-code ranging signal in the S-band downlink can be expressed as follows:

(13) $$P_{S,\mathrm{RG}}=P_{T}\cos^{2}(m_{\mathrm{TM}})J_{1}^{2}\left(m_{\mathrm{RG}}\right)$$

Under the same condition of modulation as described in Equation (9), the power ratio of the pseudo-code ranging signal relative to the total power of the downlink signal is −14.54 dB, which is also about 13% higher than that of the DOR_1_ beacon signal modulated to the downlink carrier.

Based on the analysis of Section 2 and Section 4, the measurement accuracy or exploration distance improvement of the proposed method is shown in Table 2. The scenarios are consistent with that in Table 1. As can be seen, if the exploration distance keeps unchanged, the proposed method can improve the measuring accuracy by about 5.1% and 24.6% for Scenario 2 and 3, respectively. If the RSS keeps unchanged, the exploration distance can be increased by about 6.25% and 41.25% for Scenario 2 and 3, respectively.

## 5. Results

According to the proposed method, both the S-band and X-band signals are processed using the recorded interferometry data from the CDSN to verify the combined efficacy of the pseudo-code ranging and DOR signal in this section. The S-band measured data are transmitted from China’s Queqiao-1 lunar relay satellite, and the X-band data are recoded during the static tests of a typical deep space probes.

### 5.1. Verification and Analysis of S-Band Test Data

China’s QueQiao-1 lunar relay satellite, launched in May 2018, currently resides in the Halo orbit of the second Earth–Moon Lagrange point. The S-band TT&C transponder on Queqiao-1 has implemented regenerative pseudo-code ranging and includes a set of DOR beacons. The verification of this method is facilitated by the measured VLBI data from the CDSN during the in-orbit test of the Queqiao-1 lunar relay satellite in May 2019. The T4B code with a chip rate of 2 Mchips/s is adopted for regenerative pseudo-code ranging. The test data are sampled and recorded by a single channel with a bandwidth of 16 MHz. The downlink signal spectrum design is consistent with the recommendations of CCSDS-related specifications. The two DOR tones are about ±3.8 MHz away from the main carrier. The first-order and second-order clock components of the PN code ranging signal are about 1 MHz and 2 MHz away from the carrier, respectively.

**Figure 5:**
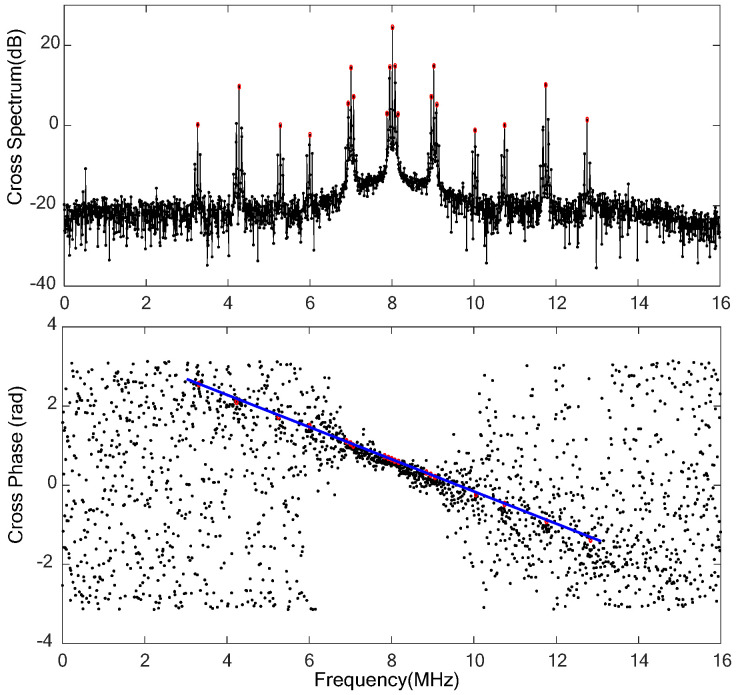
The cross-spectrum and interference fringe of the Chang’E-4 relay satellite downlink signal. The upper figure is the cross-spectrum of the downlink signal; the lower figure is the interferometry fringes (also called cross-phase).

The cross-spectrum and the corresponding fringe can be obtained by correlation processing, which are shown in Figure 5. It can be seen that the structure of the signal cross-spectrum is basically consistent with that in Figure 3. The red cycles and blue solid line in the lower half of Figure 5 indicate that the carrier and clock components of the PN code ranging signal, as well as the DOR beacon signals, exhibit clear fringes. The corresponding cross-spectrum phases are linearly distributed, which conforms to the principle described in reference [2].

**Figure 6:**
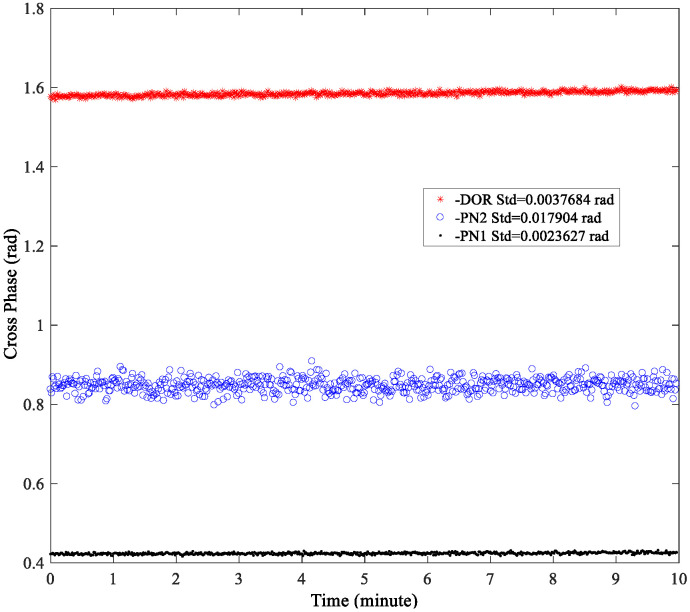
The cross-phase of several components of Chang’E-4 relay satellite downlink signal.

Figure 6 shows the cross-phase estimations of the DOR beacon signal, and the first-order and second-order clock components of the PN code ranging signal. As can be seen, the cross-phases of the three signal components are relatively stable. The accuracy of the first-order clock component cross-spectrum phase of the PN code ranging signal is the highest, being about 0.0023 rad. The cross-spectrum phase of the second-order clock component of the PN code range signal exhibits the poorest accuracy, roughly one order of magnitude lower than that of both the first-order component and the DOR beacon signal. This result is consistent with the spectrum amplitude shown in Figure 4. Considering the coupling effect between the cross-spectrum phase accuracy of the signal and the signal bandwidth, only the first-order clock component of the PN code ranging signal is used in the subsequent processing to obtain a higher coarse estimation accuracy of the time delay.

**Figure 7:**
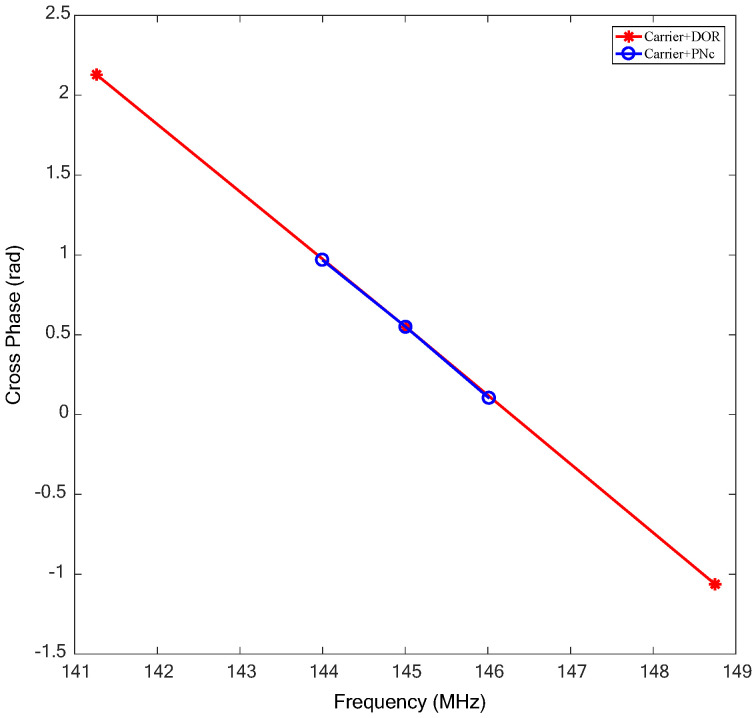
The comparison of the cross-phase of two different combinations of the downlink signal.

Figure 7 is the comparison of the cross-spectrum phase of two different combinations of the detector downlink signal. Carrier+DOR represents the traditional signal combination, namely utilizing the carrier and DOR beacon signal for frequency synchronization. The corresponding effective bandwidth is about 7.5 MHz. Carrier+PNc means the proposed signal combination utilizing the carrier and the first-order clock component of the PN code ranging signal for frequency synchronization with about 2 MHz of effective bandwidth.

**Figure 8:**
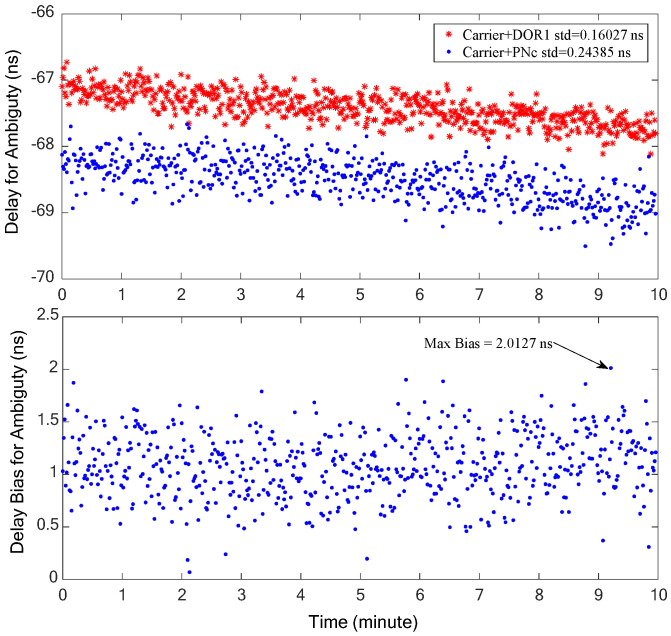
The residual delay estimation results of the two signal combinations (**upper** half) and their deviation (**lower** half).

The combination of the carrier and one DOR beacon signal, as processed traditionally, is denoted as Carrier+DOR_1_. On the other hand, the combination of the clock component of the carrier and PN code ranging signal under the proposed method is represented as Carrier+PNc. Both methods are employed for frequency synchronization, respectively, to obtain the coarse time delay estimation, thereby resolving the integer ambiguity of the ±DOR_1_ signal cross-spectrum phase. The residual delays of the two situations and their bias are shown in the upper and lower half of Figure 8. It can be seen that the trends of the time delay obtained by the two signal combinations are consistent. The accuracy of the time delay obtained by the Carrier+DOR_1_ combination is relatively higher than that of the Carrier+PNc combination. This is mainly due to the fact that the wider the effective synchronization bandwidth, the higher the accuracy of the time delay when the accuracy of the cross-spectrum phase is close. The residual delay bias obtained by the two combinations is relatively stable, peaking at approximately 2 ns, which results in a maximum bias of about 0.015 cycles (2π) and 0.08 cycles in the cross-spectrum phase when the bandwidth are about 7.5 MHz and 40 MHz, respectively. In other words, this time delay bias will not introduce the deviation of integer ambiguity resolution, and the time delay estimation results must be consistent with the traditional scheme.

**Figure 9:**
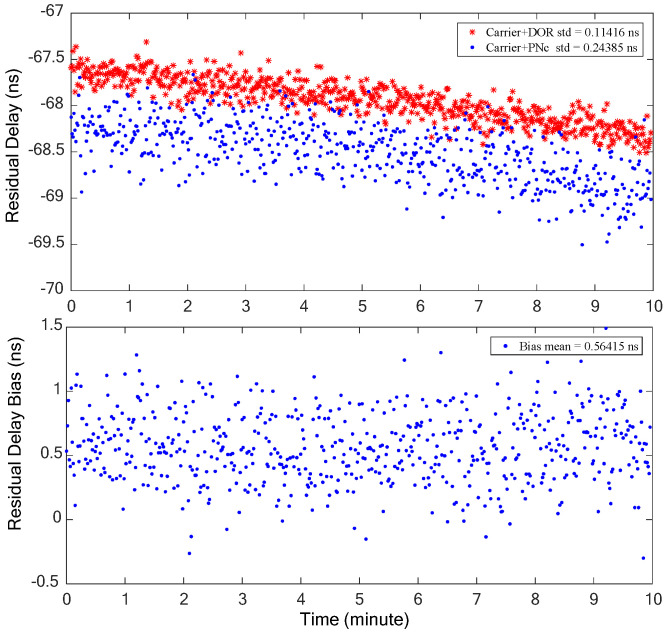
The delay estimation results of the two signal combinations (**upper** half) and their deviation (**lower** half).

Furthermore, the estimation accuracy of the interferometry time delay using only the clock component of the pseudo-code ranging signal and carrier is analyzed when the DOR beacon signal is not modulated in the S-band. The time delay is resolved by two signal combinations, as depicted in Figure 9, where Carrier+DOR represents the carrier and two DOR beacon signals, and Carrier+PNc stands for the carrier and clock components of the PN code ranging signal; therefore, the corresponding bandwidth of the effective signals are 7.5 MHz and 2 MHz, respectively. We can find that the residual time delay trends obtained by the two signal combinations are consistent with those presented in Figure 8. Notably, the residual delay random error associated with the Carrier+PNc signal combination is approximately one order of magnitude greater than that of Carrier+DOR, the discrepancy being due to the coupling effect between the bandwidth and cross-spectrum phase measurement accuracy. The deviation of the two time delay results is stable with an average value of about 0.56 ns. This may be caused by the nonlinear effect of S-band ionospheric delay, which can be corrected by differential calibration observation or ionospheric delay measurement equipment.

Considering the comprehensive influence of tropospheric delay, station location error, S-band ionospheric delay, and other sub-ns level errors, there is a marginal loss in the random accuracy of time delay (about 0.56 ns for this test scenario). Even so, the S-band signal system can still detune the DOR beacon signal, and only utilize the pseudo-code ranging clock component to achieve the interference time delay measurement. Then, the downlink signal power can be saved or the detect distance can be enlarged. This is significant for lunar and deep space exploration, especially when the downlink signal power is tight.

### 5.2. Verification and Analysis of X-Band Test Data

On the basis of the results in Section 5.1, the X-band signal system has been validated using static test data from a typical deep space probe equipped with an X-band deep space TT&C transponder. There are two groups of DOR beacon signals complying with the CCSDS specification. Meanwhile, the spacecraft adopts the regenerative pseudo-code ranging system, consistent with Queqiao-1. The spectrum of the main carrier zone under pseudo-code (T4B) and DOR tones modulation is compliant with CCSDS-related specifications. The two pairs of DOR tones are located at a distance of 3.8 MHz and 19.2 MHz from the main carrier, respectively. The frequency difference between the regenerated pseudocode ranging clock component and the main carrier is 500 kHz.

**Figure 10:**
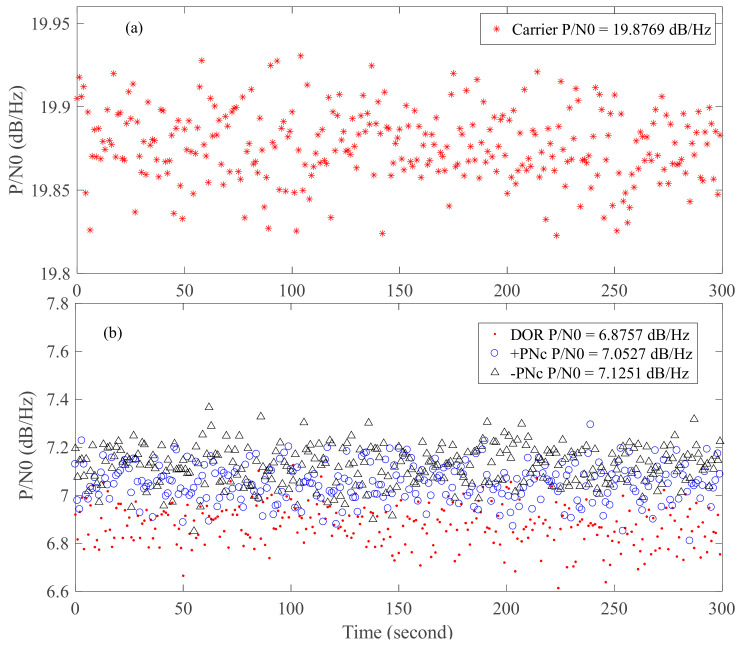
The carrier-to-noise ratio estimation results of carrier ((**a**), upper half), PN code ranging clock components (−PNc, +PNc), and DOR beacon signals ((**b**), lower half).

The carrier-to-noise ratio estimation results of the carrier, PN code ranging clock components (−PNc, +PNc), and DOR beacon signals are shown in Figure 10. It is evident that the carrier-to-noise ratios of the two PN code ranging clock components are approximately identical, at around 7 dB/Hz. The carrier-to-noise ratio of the DOR signal is about 6.8757 dB/Hz, which is close to that of the clock component of the PN code range. The carrier-to-noise ratio of the carrier signal is about 13 dB/Hz higher than that of the PN code ranging clock component.

**Figure 11:**
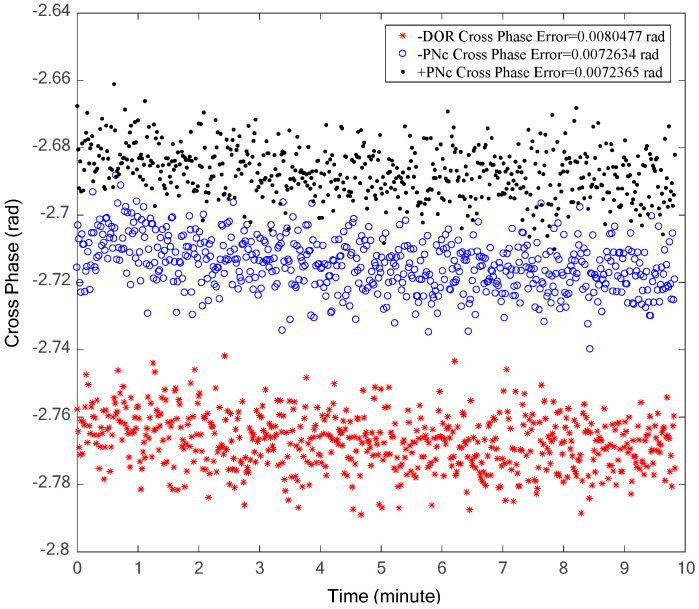
The cross-phases of DOR beacon signal or the clock component of PN code ranging signal.

Due to the relatively high carrier-to-noise ratio of the carrier signal, the random accuracy of the interference time delay primarily depends on either the DOR beacon signal or the clock component of the PN code ranging signal. The cross-spectrum phase is shown in Figure 11. The trends of the cross-spectrum phase of the three signals are consistent, with their random accuracies closely aligned. However, the random error of the DOR signal is relatively larger. This is consistent with the analyzed results of the carrier-to-noise ratio shown in Figure 10. The phase estimation accuracy of the three signals satisfies the criteria outlined in Equation (7).

**Figure 12:**
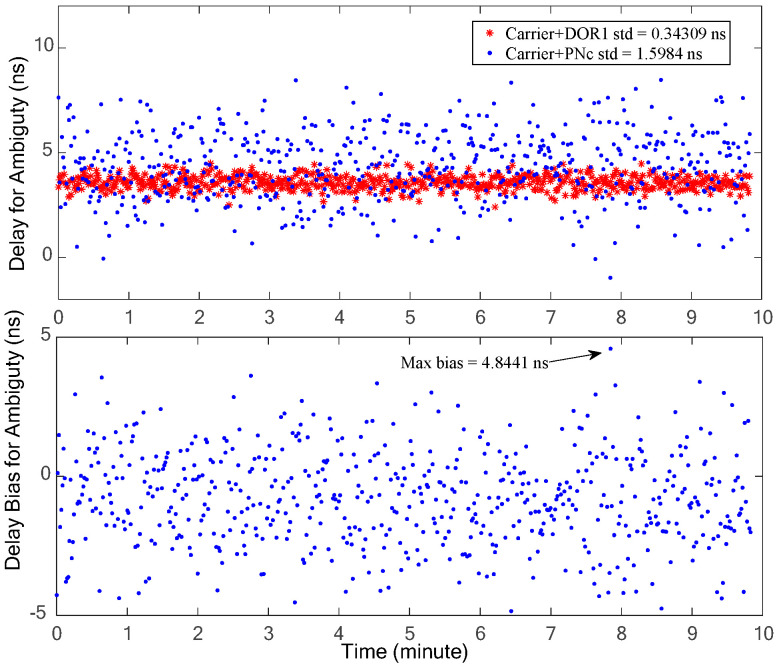
The coarse time delay estimation results of the two signal combinations (**upper** half) and their deviation (**lower** half).

In order to resolve the possible cross-phase ambiguity of ±19.2 MHz DOR beacons, two signal combinations, carrier–DOR_1_ (the traditional processing method) and carrier–PN code clock components (the proposed method in this paper), are used to estimate the coarse interferometry time delay, respectively. The coarse time delay estimation results of the two signal combinations are shown in Figure 12, in which the upper half shows the estimation results and the lower half is their deviation. It is evident that the carrier–DOR_1_ combination offers superior coarse time delay accuracy. This superiority can be attributed to the relatively larger frequency interval (namely effective bandwidth) of carrier–DOR_1_, approximately 3.85 MHz, compared to the narrower frequency interval of about 1 MHz when employing the PN code clock component. Given the equivalent random accuracy of the phase estimation, as illustrated in Figure 11, a wider effective bandwidth corresponds to the increased random accuracy of the time delay. It can be seen from the lower half of Figure 12 that the maximum deviation of the two coarse time delay estimation results is about 4.8 ns, which is almost equal to three times that of the random accuracy of the interference delay of the PN code clock component, verifying the analysis in Section 2. The cross-spectral phase deviation generated by this time delay deviation at a ±19.2 MHz DOR signal is about 0.1 cycles (2π), which does not affect the final integer ambiguity resolution result.

**Figure 13:**
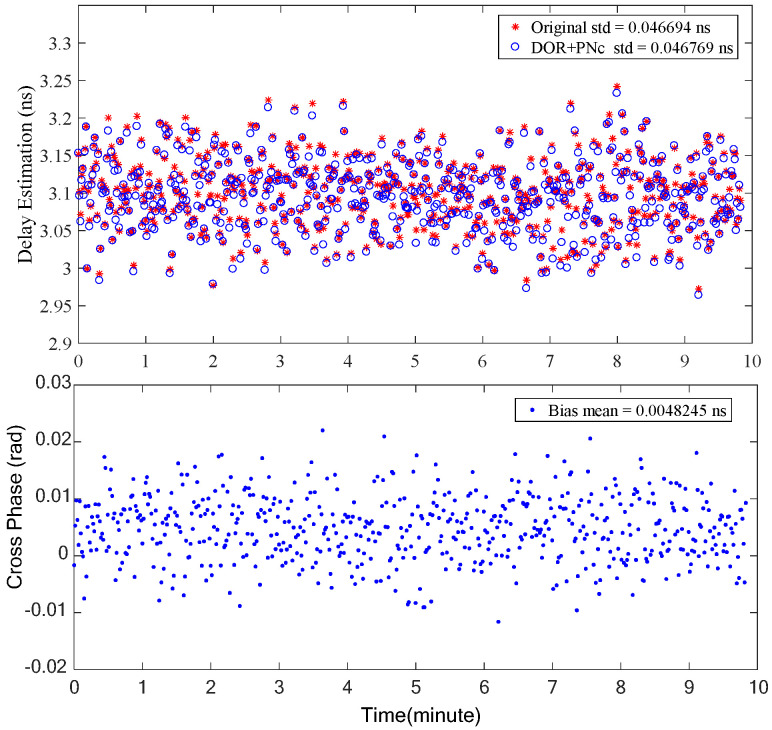
The final time delay estimation results of the two signal combinations (**upper** half) and their deviation (**lower** half).

The time delay estimation is finally achieved through a systematic ambiguity resolution process, and the results are illustrated in Figure 13. The time delay estimation results of the two signal combinations, traditional combination (denoted as ‘Original’ in Figure 13, ±19.2 MHz DOR, −3.8 MHz DOR, carrier) and proposed combination (denoted as DOR+PNc, ±19.2 MHz DOR, ±PN-Clock, carrier) are basically the same and are of close accuracy. The deviation between them is only about 5 ps. In other words, the same measurement accuracy as with the traditional signal system can be achieved by only using the DOR+PNc signal combination. Furthermore, this method transcends the traditional approach by allowing one set of DOR beacon signals to be detuned from the downlink carrier, which enhances the downlink signal power utilization efficiency or improves the deep space exploration performance. Meanwhile conversely, when the downlink signal power is tight for the X-band, the DOR_1_ beacon can be detuned and replaced by the clock component of the PN code signal for high-precision interferometry time-delay measurement in lunar and deep space exploration. 

## 6. Conclusions

This paper presents a novel joint processing interferometric measurement method, utilizing pseudo-noise ranging clock tones for integer ambiguity resolution, aimed at enhancing the power utilization efficiency of weak downlink signals in deep space exploration. First, the fundamental principles of ΔDOR are introduced and the error budget analysis is conducted. Then, the detailed analysis of several factors is provided: the power ratio of each signal component, the precision of the interferometric measurement time delay model, the measurement accuracy requirements, and the improvement of power utilization efficiency under typical downlink signal modulation parameter settings. On this basis, the on-orbit measured data of the Queqiao-1 lunar relay satellite and the static test data of a typical spacecraft were processed, analyzed, and verified. The results show that the interferometric measurement processing method combining pseudo-noise ranging and DOR beacons can achieve the measurement accuracy of traditional processing methods. If the modulation parameters of the downlink signal are maintained, approximately 13% of the X-band signal power can be saved, while the detection range can be increased by roughly 6.5%. If the power of other signal components remains unchanged, the power of the DOR signal can be increased by more than 100% to improve the accuracy of the interferometric measurements. Therefore, in future, deep space exploration missions where the probe–ground station distance is very far and the downlink signal is extremely weak, it may be feasible to adjust a set of DOR beacon signals to effectively improve the downlink signal power and its utilization efficiency. The PN code ranging clock tones can be comprehensively utilized to achieve interferometric measurement, improve the measurement accuracy, and increase the range of deep space exploration. Without additional design requirements for deep space transponders, the efficient tracking and measurement of deep space probes can be achieved.

## Figures and Tables

**Table 1:** The relative power of the downlink signal with different modulation mode.

Scenario ID	Modulation Signal	Modulation Degree	Power of Downlink Signal Relative to the Carrier
1	Telemetry signal	0.8 rad	--
	Ranging signal	0.6 rad	−15.09 dB
	DOR1 beacon signal	0.5 rad	−16.80 dB
	DOR2 beacon signal	0.5 rad	−16.80 dB
2	Telemetry signal	0.8 rad	--
	Ranging signal	0.6 rad	−14.54 dB
	DOR1 beacon signal	0.0 rad(detuned)	0 dB
	DOR2 beacon signal	0.5 rad	−16.25 dB
3	Telemetry signal	0.8 rad	--
	Ranging signal	0.6 rad	−15.09 dB
	DOR1 beacon signal	0.0 rad(detuned)	0 dB
	DOR2 beacon signal	0.7 rad	−13.60 dB

**Table 2:** Improvement by the proposed method.

Scenario ID	Spacecraft Thermal Noise	Exploration Distance	RSS	RSS or Distance Percentage	Remark
1	0.2337 ns	400 million km	0.2514 ns	100%	Reference
2	0.2199 ns	400 million km	0.2386 ns	94.91%	Distance unchanged
	0.2337 ns	425 million km	0.2514 ns	106.25%	RSS unchanged
3	0.1655 ns	400 million km	0.1896 ns	75.42%	Distance unchanged
	0.2337 ns	565 million km	0.2514 ns	141.25%	RSS unchanged

## Data Availability

This study used the measured data from Chinese Deep Space Stations, but according to the copyright, we cannot afford an open dataset. But the method proposed here is general, and can be applied to process any data which have the PN Clock component and DOR tone signals.
